# ProMiner: rule-based protein and gene entity recognition

**DOI:** 10.1186/1471-2105-6-S1-S14

**Published:** 2005-05-24

**Authors:** Daniel Hanisch, Katrin Fundel, Heinz-Theodor Mevissen, Ralf Zimmer, Juliane Fluck

**Affiliations:** 1 Fraunhofer Institute SCAI, Schloss Birlinghoven, 53754 Sankt Augustin, Germany; 2 Current address: Aventis Pharma Deutschland, Industriepark Hoechst G879, 65926 Frankfurt am Main, Germany; 3 Institute for Informatics, Ludwig-Maximilians-Universität München, Amalienstrasse 17, 80333 München, Germany

## Abstract

**Background:**

Identification of gene and protein names in biomedical text is a challenging task as the corresponding nomenclature has evolved over time. This has led to multiple synonyms for individual genes and proteins, as well as names that may be ambiguous with other gene names or with general English words. The *Gene List Task *of the BioCreAtIvE challenge evaluation enables comparison of systems addressing the problem of protein and gene name identification on common benchmark data.

**Methods:**

The ProMiner system uses a pre-processed synonym dictionary to identify potential name occurrences in the biomedical text and associate protein and gene database identifiers with the detected matches. It follows a rule-based approach and its search algorithm is geared towards recognition of multi-word names [1]. To account for the large number of ambiguous synonyms in the considered organisms, the system has been extended to use specific variants of the detection procedure for highly ambiguous and case-sensitive synonyms. Based on all detected synonyms for one abstract, the most plausible database identifiers are associated with the text. Organism specificity is addressed by a simple procedure based on additionally detected organism names in an abstract.

**Results:**

The extended ProMiner system has been applied to the test cases of the BioCreAtIvE competition with highly encouraging results. In blind predictions, the system achieved an F-measure of approximately 0.8 for the organisms mouse and fly and about 0.9 for the organism yeast.

## Background

The correct identification of biological entities in scientific text is a fundamental requirement for information retrieval and information extraction in the biomedical domain. Using the extracted identifiers, facts from the literature can be efficiently organized, e.g. in the form of a graph [2], and linked to experimental data, such as expression measurements.

The task of biological name identification is difficult as the employed nomenclature is highly variable and ambiguous. During the long history of biomedical research, the use of phenotypical descriptions as protein names, the definition of gene aliases as convenient abbreviations of corresponding protein names and the parallel naming of genes and proteins have all influenced the nomenclature. Consequently, multiple synonyms often exist for the same entity. Identical names are used to identify different biological entities depending on their context and, in some cases, a significant overlap between gene names and common English words exists. Every organism has its own research history which is reflected in the nomenclature. The yeast organism, for example, has a relatively stringent nomenclature. Mouse proteins often have several similar, slightly varying multi-word names. The fly organism poses the most difficult problem due to the significant overlap of fly gene names with common English words. Furthermore, a number of gene names differ only in their case.

Several systems address the problem of name detection without identifying the corresponding protein and gene identifiers. This is accomplished using features mainly derived from lexical considerations and natural language processing techniques such as part-of-speech tagging. Methods vary in the details of feature computation and their combination. Rule-based approaches [3,4] as well as machine-learning based techniques [5,6] have been proposed. Task 1A of the BioCreAtIvE challenge evaluation addresses this problem [7,8].

The arguably more difficult task of also identifying the corresponding protein and gene name identifiers was the focus of Task 1B of BioCreAtIvE [9]. It has been discussed in the literature to a lesser extent. The first suggestion in that domain was to treat protein name detection as an alignment problem and use the BLAST algorithm to efficiently solve it [10]. Jenssen et al. [2] composed a dictionary of gene names and used simple string matching for name detection to annotate microarray data. We proposed the ProMiner system [1] which consists of two parts. First, a dictionary is generated from several protein and gene databases. As the name and synonym fields in these databases often contain physical descriptions (*cDNA clone*, *RNA*, *5'end*), family names (*membrane protein*) or other annotation remarks, the dictionary is cleaned in an automated process. Based on this dictionary, a string match procedure is used for name detection in the text. The system was initially designed for human protein and gene names, which often consist of multiple words. In that setting, name variants, e.g. permutations, insertions or deletions of words, were observed. For example, the name "Interleukin type 1 beta" is a spelling variant of "Interleukin-1 beta". However, "Interleukin 1" is a different protein entity than "Interleukin-1 receptor". The ProMiner match procedure assigns weights to different classes of words to reflect their importance for name detection. These are termed *token classes*. The complete system is described in more detail in the Implementation section. Besides customization for the BioCreAtIvE evaluation, extensions for synonym classification, match disambiguation, and organism specificity have been added.

The original ProMiner system was tested on a small benchmark set of Human gene and protein names, showing promising performance. Due to the lack of large, independent benchmark sets, comparisons to other systems were difficult. The Gene List Task (Task 1B) of the BioCreAtIvE challenge evaluation provides such evaluation data sets and, for the first time, enables researchers to evaluate and compare their protein and gene name recognition systems in blind predictions. The task focused on three organisms (fly, mouse, and yeast) for each of which a training and a test data set was provided. After the training phase, each participant had to submit blind predictions for an independent set of 250 biomedical abstracts. After assessment of the results, the two measures of *precision *and *recall*, and a balanced combination of these, the *F-measure*, have been reported by the task organizers to compare the performance of the submissions. Furthermore, the gold standard for the data set was published for error analysis. A detailed description of the task and all involved data sets is given in [9].

## Implementation

The extended ProMiner system consists of three parts: dictionary generation, occurrence detection and filtering of matches. The first part covers the generation and curation of a name dictionary, which associates each biological entity with all known synonyms. As part of this step, each synonym is classified into one of several classes which are associated with specific parameter settings in the subsequent search runs. The second part of the system consists of an approximate search procedure which is geared towards high sensitivity. It accepts different parameter settings for each of the synonym classes. This procedure is applied to detect all potential name occurrences on basis of the constructed dictionary. For disambiguation of found protein and gene name matches, names from external dictionaries are detected in the text. These external dictionaries contain acronyms, organism names, cell types, and other biological entities. In a last step, filters are applied to increase specificity of the search results. The disambiguation filter attempts to resolve ambiguous matches. Such matches exist because two or more proteins might share a synonym, acronyms are common in different contexts, or synonyms are also used as regular English words. Finally, an organism filter uses co-occurring matches of organism names to accept or reject organism-specific name occurrences.

The ProMiner system treats each synonym as a string of letters which can be split into several *tokens*. These tokens generally corresponds to words or numbers. For instance, the string "interleukin-1 receptor" would be split into four tokens: "interleukin","-","1","receptor". The detection problem is addressed on the level of such tokens. Tokens are equivalent if their strings match exactly. Depending on the parameter setting, the case of the strings has to match as well. Furthermore, the set of all tokens is categorized according to *token classes *which vary in significance for occurrence detection. For example, the token "receptor" is of higher relevance for a match than the token "type".

### Dictionary generation: construction and curation

In Task 1B of the BioCreAtIvE competition, dictionaries for each test organism were provided defining the set of objects for detection. The ProMiner dictionaries for *mouse *and *yeast *were created on the basis of these provided lists. The *fly *dictionary was obtained directly from the FlyBase  database due to format problems with the provided list. Entries were explicitly limited to *D. melanogaster *genes. All dictionaries have been processed using the following steps. First, subtype specifiers are expanded to equivalent other specifiers, e.g. *a *is expanded to *alpha*. Similarly, common acronyms are expanded to their equivalent long forms, e.g. *IL *is treated equivalently to *Interleukin*. Spaces are inserted or removed between words and digits, e.g. *Igf1 *and *Igf 1 *are considered equivalent. In a next step, all synonyms of the dictionary are tagged with a corresponding string of token classes. Regular expressions defined on these token classes allow for detection of unspecific synonyms. For example, let *M *describe the token class subsuming all measurement units and let *d *be the token class of all numeric tokens. Then a regular expression of the form d* M would detect all synonyms consisting of an optional number and a measurement unit, such as *22 kDA*. The detected synonyms are either pruned from the dictionary (mouse) or marked for later processing (fly, yeast). Furthermore, manually derived lists of family names and physical descriptions are also used to filter out unspecific synonyms.

These general curation steps are followed by organism dependent ones. In yeast, the only modification was the addition of the letter 'p' to each gene name. For the fly dictionary, we manually removed 10 synonyms and added 6 synonyms from specific dictionary entries on the basis of the training set. The curation steps for the mouse dictionary are described in Fundel et al. [11]. In that work, name detection is accomplished using a simpler matching procedure. Therefore, the curation is geared towards generation of a comprehensive dictionary including many feasible spelling variants and strict removal of unspecific synonyms.

### Dictionary generation: rule-based classification of synonyms

For each pre-processed dictionary, a partition of the set of synonyms into three classes is determined. First, frequencies of occurrence of all stemmed words in all abstracts of the MEDLINE database are computed using the Porter stemmer [12]. Based on this data set, all frequently occurring one-word synonyms are assigned to the class of *unspecific synonyms*. The rationale is that frequently occurring words are unlikely to be used as protein names only. The frequency threshold was estimated based on the training set for the fly organism and remained unchanged for the other organisms. In addition, synonyms detected via the regular expressions in the previous curation steps are assigned to this class. Synonyms in the class of *unspecific synonyms *are used for disambiguation of other matches only. In addition, these synonyms are augmented with specific context words (e.g. protein, gene, transcripts) and added as additional synonyms. Context words are appended to ensure that a synonym occurrence refers to a gene or protein name, e.g. the detection of the fly dictionary name *clipped *alone would not result in a match, the detection of the term *clipped gene *would. In the search run, only exact matches are accepted for these augmented synonyms.

Synonyms which must be searched in a case-sensitive manner constitute the class of *case-sensitive synonyms*. If a synonym can only be distinguished from a synonym of another dictionary entry when the case of the letters is considered, it is assigned to the class of *case-sensitive synonyms*. This is tested with respect to names from the protein dictionaries as well as external dictionaries, e.g. containing anatomical terms.

All other synonyms are part of the class of *case-insensitive synonyms *and are detected in the text using an approximate matching procedure in a case-insensitive manner.

### Occurrence detection: efficient gene and protein name detection

**Figure 1:**
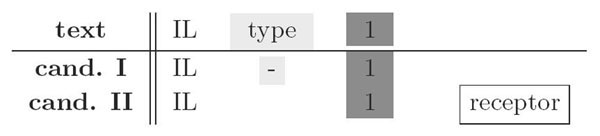
**First example of impact of token classes. **Candidate synonym I is a correct synonym match, whereas candidate II is not. Appropriate weighting of tokens allows to detect the differences correctly.

**Figure 2:**
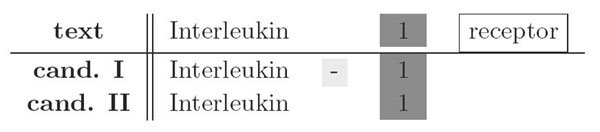
**Second example of impact of token classes. **Both candidates are wrong matches because the significant token "receptor" is present in the text. Naive matching would accept both candidates.

The search procedure [1] works by sweeping over the abstract, processing one token at a time and keeping a set of candidate solutions for the present position. Each candidate solution is associated with two scoring measures. One scoring measure, the *boundary score *controls the end of the extension of a candidate match and is increased on a token mismatch. If this score rises above a threshold, i.e. if a certain number of mismatches has occurred, the candidate is pruned from the candidate set and checked for reporting. Then, the second score measure, the *acceptance score*, determines whether the candidate is reported as a match. The acceptance score is a linear combination of token class specific match- and mismatch terms. A *match term *is defined as the *percentage of matched tokens *of the respective token class. A *mismatch term *counts for each token class the *number of tokens additionally found in the text during this candidate extension *and, thus, mismatched in the candidate synonym. With appropriate weighting, the acceptance score makes it possible to accept variations of synonyms and, at the same time, disregard false substring matches. To illustrate this, consider the example depicted in Figure 1. Candidate synonym I is a correct synonym match, whereas candidate II is not. Exact string matching would find none of the candidates. In both cases, one token in the text excerpt ("type") and one token of each candidate is not matched. The situation can be distinguished if weights for match terms are set to a small value for non-descriptive tokens, such as "-" or "type" and a high one for modifier token, such as 'receptor'. Now consider a reversed situation as shown in Figure 2. Here, both candidates are wrong matches because the significant token "receptor" is present in the text. Naive matching would accept both candidates. Setting the mismatch weight for the modifier class to a high value, however, will lead to the rejection of both candidates.

Synonyms can be treated differently, depending on their synonym class. In the *exact search*, which is used for *unspecific synonyms*, no deletion, insertion or permutation of tokens is allowed. The *approximate search *allows for deletion, insertion or permutation of tokens if the synonym consists of more than 2 tokens. Depending on the class of the synonym, the search is executed in a *case-sensitive *or *case-insensitive *manner. The weighting of the acceptance score components has remained unchanged and is based on the small benchmark set as described in [1]. Generally, it penalizes the deletion or insertion of modifier token such as "receptor" heavily and allows the deletion and insertion of non-descriptive tokens such as "-" or "type".

### Occurrence detection: inclusion of external dictionaries

For match disambiguation, we detected occurrences according to additional external dictionaries containing biological processes and cellular component names from the Gene Ontology , fly body parts from FlyBase  and cell types. In addition, an abbreviation dictionary containing abbreviations and their long forms, which do not correspond to protein or gene names, is compiled from two sources. First, we extracted short uppercase synonyms from the employed dictionaries and queried the Biomedical Abbreviation Server [13] for potential long forms. Secondly, we extracted from all abstracts all short expressions in parentheses as possible abbreviations of long forms mentioned directly ahead of the parentheses, e.g. "... respiratory distress syndrome (RDS) ...". An abbreviation is accepted, if for each letter in the abbreviation a corresponding word in the preceding expression is found. A similar approach has been described in [14]. Finally, the long forms of all abbreviations are checked against each protein and gene name dictionary in order to prune long forms which correspond to protein names of the considered organism.

### Filtering of matches: match disambiguation

The search procedure based on all constructed dictionaries results in a set of synonym occurrences for each biomedical abstract. If only one corresponding identifier is detected at a certain position in the text, the match is accepted. If matches overlap, the match with the higher acceptance score, the larger fraction of matches or the largest number of matched tokens is accepted. In conjunction with the controlled vocabulary, this leads to the removal of unspecific synonyms. For example, the gene *furrow *is often found as a substring match of the term *morphogenetic furrow *describing a fly body part. As the second term will attain a higher score, it will be accepted, preventing the erroneous detection of the gene name.

In case of an ambiguous synonym that is used for two or more different genes or proteins, only those matches are accepted for which most additional synonym occurrences can be found in the abstract. A threshold defining a maximal number of resulting ambiguous matches in one position can be set and has been varied in the three submitted runs of the competition. This parameter is named 'D#' in the Results section, where '#' stands for the maximal number of ambiguous matches.

### Filtering of matches: organism filter

To account for required organism specificity of matches, we constructed a filter based on the NCBI taxonomy (NCBI Taxonomy database, ). Organism name occurrences were detected using exact matching in the given text corpus. For each search run, a set of relevant organisms has to be defined. Basically, abstracts are rejected if they contain only organism names currently considered irrelevant or their generalizations in the taxonomy. If only relevant organism names or their generalizations are found, the abstract is accepted and all detected protein and gene occurrences reported. In cases where relevant and irrelevant organism names occur, the decision is left to the user. For the competition, we chose to accept abstracts in those cases. As an example, consider a search run for *D. melanogaster*. An abstract mentioning only the organism name 'D. yakuba' would be rejected. An additional occurrence of the concept 'Drosophilae' in the abstract would also lead to rejection, as this is a generalization of 'D. yakuba' in the taxonomy. In contrast, an abstract mentioning the term 'Drosophilae' only would be accepted, as this is also a generalization of the relevant organism *D. melanogaster *and no evidence of another more specific organism was found. This simple strategy will fail if genes and proteins from diverse organisms are discussed in one abstract, requiring more involved analysis of the reference structure in the abstract. However, in the context of the BioCreAtIvE competition, it improved the performance for the fly organism significantly.

## Results and Discussion

We computed three search runs for each organisms which differed with respect to the intended precision-recall tradeoff (cf. Table 1). The values of three parameters were changed.

The *Disambiguation threshold *(D#) controls the size of the result set, when ambiguous synonyms are detected. If the size of the result set exceeds this threshold after application of the disambiguation filter, none of the putative name occurrences will be reported. The *use of the organism filter (O+/O-) *should increase precision of matches. However, performance did not improve for the mouse organism based on the training set. *Significance of '-' at the end of a synonym *(S+/S-) can prevent erroneous matches such as '*protein name *– induced'. Here, S+ disallows matches ending in a dash, S- denotes that a dash can be ignored.

The results obtained with the ProMiner approach are also summarized in Table 1. Overall, our approach received highly encouraging results. For the mouse organism as well as for the fly organism, the best F-measures of all participants were obtained. The yeast results were also above average, although we did not extend the dictionary provided by the task organizers with yeast protein names. Presumably, such an extension could further increase performance for the yeast organism.

### Analysis of results

The published gold standard makes it possible to determine the impact of the various components of the ProMiner approach. The evaluation of the different parameter settings in the three different organisms shows that the best results are achieved setting the disambiguation threshold to one (D1) and treating a dash as significant (S+). Not in all cases was the best possible parameter combination submitted, however. In general, the disambiguation threshold D1 leads to higher precision with a minor loss in recall. The influence of the dash on the F-measure is not clear, however. The impact of the organism filter differs for the organisms fly and mouse. In the case of fly, the filter enhances precision without incurring a loss in recall, for the mouse data, however, the filter reduces recall without gaining precision. Possible reasons for that are discussed in the mouse section. Analysis of results with respect to each organism is presented in the following.

### Fly

For fly, all three submitted ProMiner runs outperform the results of the other participants. The best result was achieved setting the disambiguation threshold to one (D1), enabling the organism filter (O+) and treating a dash as significant (S+).

**Figure 3:**
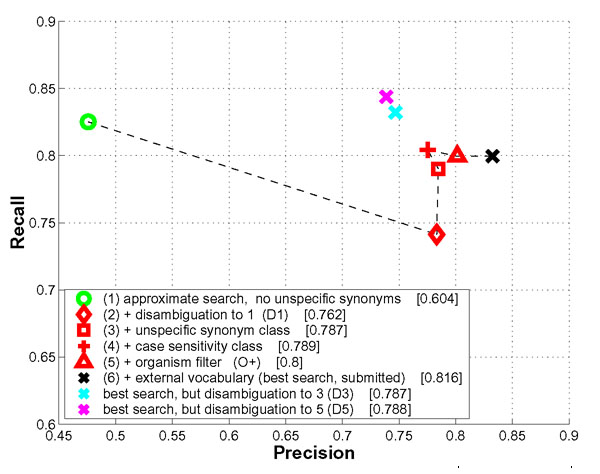
**Impact of ProMiner components for the fly organism. **For each result, the F-measure as determined from the published gold-standards is given in brackets. Details on this figure are provided in the *Results and Discussion *section.

The fly organism probably poses the most severe problems to name detection as common English words occur frequently as parts of protein/gene names. Figure 3 shows results for different parameter settings which have been computed after the gold standard for the test set was released. Naïve string search with the provided dictionary is infeasible for fly and will result in unacceptable precision (approximately 0.06, data not shown). The detection of unspecific synonyms will alleviate this problem. A search based on the ProMiner core algorithm using only synonyms not in the unspecific class and no further disambiguation leads to high recall but low precision ('approximate search, no unspecific synonyms'). Using the maximal disambiguation strategy with a threshold of one already provides acceptable performance (F-measure: 0.762, '+ disambiguation to 1 (D1)'), although the recall is decreased. The incorporation of unspecific synonyms improves recall considerably (F-measure: 0.787, '+ unspecific synonym class'). The additional detection of case-sensitive genes improves recall further, but impacts precision (F-measure: 0.789, '+ case sensitivity class'). An increase in recall is observed as previously ambiguous occurrences can now be assigned to one synonym with the correct case. However, false positives also occur in this set decreasing precision. The organism disambiguation for the fly organism seems to work well as only subspecies differentiation was needed in the test set (F-measure: 0.80, '+ organism filter (O+)'). A final gain in precision is obtained through incorporation of external dictionaries for disambiguation purposes. This results in the submitted run with maximum F-measure (F-measure: 0.816, '+ external vocabulary (best search, submitted)').

For comparison, the disambiguation thresholds D3 and D5 (F-measure: 0.787/0.788, 'best search, but disambiguation set to D3/5') negatively impact precision and recall. A-priori this was unclear as the training set contained examples, where (1) different allelic variations of the same gene or (2) members of a complex were annotated as true positives. These cases profit from a higher disambiguation threshold. In the test set, however, the *disambiguation to one *strategy (D1) provided the best results.

Concluding, all components of the ProMiner framework improve overall performance to various degrees. The most important steps for the fly organism are the detection and sensible incorporation of unspecific synonyms and the 'disambiguation to 1 (D1)' setting.

### Yeast

The results for yeast exhibit good performance for submitted runs of most participants reflecting the quite stringent terminology followed for that organism. In this setting, simpler name recognition approaches seem to suffice to obtain satisfactory results. Probably, the incorporation of multi-word protein names into the synonym list would improve recall for our approach. Such improvements should be pursued in future work.

### Mouse

The mouse nomenclature is not dominated by common word names as for fly nor is it as stringent as in the case of yeast. Multi-word protein names are frequent for the mouse organism. In general, overall results of all participants are better than for the fly organism but worse than for yeast. The ProMiner submissions achieved the best F-measures.

**Figure 4:**
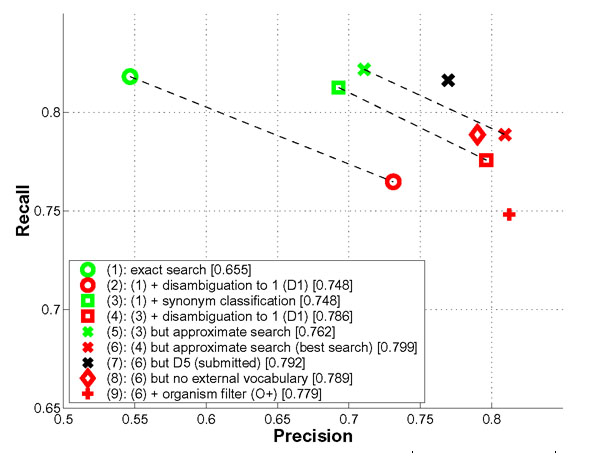
**Impact of ProMiner components for the mouse organism. **For each result, the F-measure as determined from the published gold-standards is given in brackets. Details on this figure are provided in the *Results and Discussion *section.

A detailed analysis of the ProMiner approach is given in Figure 4. All runs were computed without organism disambiguation (O-) and treating a dash as significant (S+), if not stated otherwise. For mouse, an exact, case-insensitive search and no further disambiguation is feasible, although results are mediocre (F-measure: 0.655, 'exact search'). In this setting, no insertion, deletions or permutations of tokens were allowed. Disambiguation to one allowed object impacts recall, but increases precision to acceptable values (F-measure: 0.748, '(1) + disambiguation to 1 (D1)'). The incorporation of case-sensitive and unspecific synonyms improves both precision and recall (F-measure: 0.786, '(3) + disambiguation to 1 (D1)'). A final gain of overall performance stems from the approximate search as described in the Implementation section (F-measure: 0.799, '(4) but approximate search'). This last gain in performance is only slight. This might indicate that spelling variants of multi-word names not contained in the dictionary were not frequent in the BioCreAtIvE test set. This optimal search run was not submitted, however, because the impact of the organism disambiguation procedure was unclear from the training set. Using the same search parameters including organism disambiguation leads to worse recall (F-measure: 0.779, '(6) + organism filter (O+)'). The first thirty false positive matches of the best submitted result for mouse (run number 3) have been analyzed in more detail (cf. Table 2). Undetected ambiguities are the dominant reasons for false positive matches (18 cases, 60%). This includes synonyms which have neither been detected during curation nor marked as unspecific synonyms, synonyms detected in wrong contexts and abbreviations unknown to the system. Erroneous detection of genes from other organisms accounts for 4 false matches (13%). In eight cases (27%), the reason for exclusion from the gold-standard remained unclear.

To assess the impact of the stringent dictionary curation procedure applied in the case of mouse [11], we computed the performance of the ProMiner system based on the dictionary as provided by the task organizers. Interestingly, the classification of synonyms into different classes and the associated disambiguation strategy were sufficient to achieve a good result. Using the parameters leading to the best result for the cleaned dictionary (D1, O-, S+; F-measure 0.799) gives an F-measure of 0.78 (data not shown). This indicates that the ProMiner framework provides good performance even on dictionaries of medium quality without manual curation due to its automatic detection of unspecific and case sensitive synonyms. An analysis of the organism disambiguation performance revealed that the disambiguation failed when genes from different organisms are mentioned in one abstract. Also, disambiguation failed because of missing synonyms, e.g. "vertebrate". For several cases, however, the provided gold standard might be incorrect as occurrence considered as true positives describe findings in rat or human instead of mouse. For a more comprehensive evaluation of such disambiguation strategies, a more specific benchmark set is required.

In conclusion, the ProMiner method provides high recall using approximate matching and retains high precision due to sensible incorporation of unspecific synonyms for mouse protein and gene name recognition.

### Discussion and future work

Further reduction of false positive matches might be achieved with a sensitive prior tagging of all putative protein occurrences in a given text as a first step, and assignment to objects from a dictionary in a second step. The best participants in Task 1A, concerned with name tagging only, obtain a recall and F-measure of about 0.83 [7]. This level of performance might allow slight improvements for Task 1B. One group, pursuing this strategy and participating in both tasks, reached high precision but considerably lower recall in Task 1B [15]. This indicates that either a higher recall in the tagging step is necessary or the assignment of objects to tagged names needs to be improved to reach better overall performance. In future work, the performance of the ProMiner framework will be tested as part of such a strategy.

Highly sensitive detection of the dictionary synonyms and subsequent filtering of false positives on the basis of machine learning methods seems also feasible. This approach led to good results even for the fly organism [16]. While the rule-based ProMiner system can be adapted quickly to a new settings by human experts, a machine-learning based approach requires a comprehensive training set of high quality to obtain good results. If such training sets become readily available through a community effort, however, machine-learning based approaches might be superior to rule-based systems.

Another approach to solve the name recognition problem is the generation of a comprehensive and stringently cleaned dictionary, which contains many spelling variants. This dictionary can be used in conjunction with a simpler matching procedure [11]. This strategy works well for the mouse and yeast organisms, but is not feasible for organisms with high prevalence of unspecific names, such as fly. The introduction of post-filters might render this approach feasible in most cases. In its current form, the approach seems to rely even more on expert curation than the ProMiner framework. Also, the size of the dictionary might grow quickly, if automatically generated spelling variants are included, which might adversely impact the running time of the search.

## Conclusion

The named entity recognition task of the BioCreative challenge was ideally suited for independent evaluation of the ProMiner method. The task required the customization of the framework to the organisms of fly, mouse and yeast, each of which exhibits specific naming characteristics. After incorporation of the extensions, the ProMiner method could be quickly adapted to the characteristics of each organism using parameter settings and customized dictionary curation. For the mouse organism, the comparison with a simpler name matching procedure [11] has shown that the same level of performance can be reached when the dictionary used is cleaned more stringently. The ProMiner system was able to obtain a high level of performance based on the classification of synonyms into several search classes. The results of the competition were highly encouraging and indicate that approximate matching in conjunction with rule-based pre- and post-processing is well suited for protein and gene name recognition. The ProMiner method obtained F-measures of 0.899 for yeast, 0.816 for fly, and 0.79 for mouse.

Interestingly, the organizers estimated the quality of the initial gold standard by comparing annotations provided by different experts. On a sample of 30 abstracts, the disagreement was 9% for yeast, 13% for fly, and 31% for mouse [17] reflecting the different degree of uncertainty of nomenclature in the various organism. According to these results, the automatic annotation methods already achieve a level of performance comparable to the initial annotations by biological experts.

Given the good performance of the ProMiner framework in the domain of protein and gene name detection, a venue of future work will be its extension to other biomedical entities. Adaptation of the system to the recognition of phenotypic descriptions or functional categories is a next step in the development of an application which efficiently detects a multitude of biologically relevant named entities in biomedical text.

## Figures and Tables

**Table 1:** Results of the BioCreative competition. Summary of submitted search runs. The table contains details of parameter sets (D# denotes disambiguation threshold, O(+/-) use of organism disambiguation, S(+/-) significance of dash at end of synonym) and resulting performance. Values where ProMiner achieved the best rank of all participants are printed in **bold**.

	Fly			Mouse			Yeast	
	1	2	3	1	2	3	1	2
Disambiguation (D#)	D3	D1	D3	D3	D1	D5	D3	D1
Organism O(+/-)	O+	O+	O+	O-	O+	O-	O-	O-
Dash significance S(+/-)	S-	S+	S+	S-	S+	S+	S-	S-
F-measure	0.781	**0.816**	0.787	0.771	0.776	**0.79**	0.897	0.899
Precision	0.728	**0.831**	0.744	0.752	0.809	0.766	0.951	0.966
Recall	**0.841**	0.8	0.834	0.79	0.746	0.814	0.848	0.84

**Table 2:** Examples for false positive matches in the BioCreative competition. Samples of false positive matches in the best submitted mouse search run. Detected matches are printed in **bold**.

Description	Examples
Unspecific synonym	**growth retarded**, **perinatal lethality**, **long lived**
Wrong context	**TGF-beta **superfamily, **c-myc **tumors
Unknown ambiguity	high dose set at MTD or **MFD**
Unclear, why marked as incorrect	**interleukin-2**, **H-2 **locus, **c-Jun**
